# Honor-Based Abuse in England and Wales: Who Does What to Whom?

**DOI:** 10.1177/1077801220952168

**Published:** 2020-08-31

**Authors:** Lis Bates

**Affiliations:** 1 Open University, Milton Keynes, UK

**Keywords:** honor-based violence/abuse, forced marriage, domestic violence/abuse

## Abstract

Key findings are presented from an empirical study profiling 1,474 cases of honor-based abuse (HBA) known to police and victim services in England and Wales. Thematic and quantitative (regression) analyses were used to investigate whether and how HBA differed from other forms of domestic abuse and forced marriage. A new typology of HBA is proposed, based principally on the relationship(s) between victim and perpetrator(s). Interpreted within an overarching lens of gender-based violence, it is argued that Type 1 (partner abuse) and Type 3 (partner plus family abuse) are culturally specific forms of domestic abuse, whereas Type 2 (family abuse) is distinct.

## Introduction

The lack of comprehensive data makes it difficult to understand [honor-based abuse] and formulate appropriate policy responses.

—Parliament, House of Commons (2008, p. 19).

So concluded the U.K. Parliament’s Home Affairs Select Committee inquiry into honor-based violence in 2008. The same lack of empirical evidence formed the basis for the research study on which this article draws. I suggest that, through large-scale analysis of incidents identified as honor-based abuse (HBA) in England and Wales, we can reach a nuanced understanding of the nature of abuse and who is involved.

In England and Wales, over the past 20 years, government policy has increasingly responded to specific forms of HBA, particularly forced marriage (now a criminal offense) (Her Majesty’s Government [HMG], 2014). There has been media coverage of the highest risk and highest profile cases of so-called “honor killings” (e.g., the cases of Banaz Mahmood in London and Rucksana Naz in Derby; Siddiqui, 2014). Such cases have raised the political and public profile of HBA but, at the same time, have “exoticized” it as something “other,” polarizing it from mainstream forms of domestic and intimate partner abuse (Thiara & Gill, 2010). The rest of the spectrum of HBA (anything short of an “honor killing”) has been overshadowed (Aplin, 2017; Idriss, 2017). Insufficient scrutiny, especially using empirical evidence, has been brought to bear on the following: what behavior is involved in HBA cases which do not involve murder, whether there are specific patterns or forms or combinations of abusive behavior in these cases, and—in particular—who, and how many, individuals in the family or community are doing the abuse.

A further complication arises from the frequent conflation of HBA with forced marriage and female genital mutilation (FGM) (e.g., Her Majesty’s Inspectorate of Constabulary [HMIC], 2015, which combined these abuses together with HBA). Conceptually, forced marriage, and to a lesser extent FGM, both relate to ideas of honor, shame, and the currency of female sexuality. However, the amalgamation of these two specific practices with HBA has meant that forced marriage and FGM are more frequently the focus of research, law, and policy, compounding a lack of understanding of the nature of other cases of HBA.

In terms of research, the nature of HBA means that—like other forms of interpersonal violence—it is often “hidden.” Empirical studies have used qualitative methods such as interviews or focus groups with victims (often using gatekeeping organizations to arrange access; see Hester et al., 2008, 2015, on forced marriage), or interviews with professionals responding to cases (e.g., Begikhani et al., 2015; Idriss, 2017). Other work has been principally theoretical (Chantler & Gangoli, 2011). This is particularly true when studies which focus wholly or exclusively on forced marriage are excluded. Examinations of HBA cases have typically been qualitative and have rarely involved large sample sizes. University of Bristol research conducted for HMIC in 2015, involving 60 victim interviews, was one of the larger qualitative sample sizes (Hester et al., 2015). Quantitative studies (e.g., HMIC, 2015, reporting data on police-flagged HBA crimes and incidents; or annual Crown Prosecution Service [CPS] data publications on cases of HBA prosecuted in England and Wales), have larger sample sizes, but only offer a snapshot of cases known to a particular agency at a particular point in time, and only measure limited precoded variables. Challenges in understanding and responding to HBA arise from (a) a lack of systematic scrutiny of cases, and (b) a lack of distinct research on HBA overall (as opposed to specific forms of abuse such as forced marriage or FGM). This article approaches these knowledge gaps through systematic examination of 1,474 HBA cases in England and Wales identified by police and specialist services supporting victims. In the context of cases known to these agencies, it addresses the following two research questions:

**Research Question 1 (RQ1):** Who does what to whom in cases of HBA?
**Research Question 2 (RQ2):** How is HBA similar to or different from other cases of domestic and intimate partner abuse?

## Background

### Defining and Framing HBA

There is no statutory definition of HBA in England and Wales (and it is not a specific criminal offense), but a common definition has been adopted across government and criminal justice agencies: “a crime or incident which has, or may have been, committed to protect or defend the honor of the family and/or community” (Crown Prosecution Service, n.d.).^1^

Forced marriage is a criminal offense in England and Wales, and it is defined as “a marriage in which one or both spouses do not consent to the marriage but are coerced into it” (HMG, 2014, p. 5). In policy and conceptual terms, it is often treated interchangeably with HBA, along with FGM (e.g., HMIC, 2015; National Police Chiefs’ Council, 2015). Forced marriage often occurs in the context of HBA, sometimes occurring as a “corrective” to perceived dishonorable behavior, such as being gay or choosing a partner the family considers unsuitable (Samad, 2010). It can also be a trigger for HBA, especially where a child rejects a spouse chosen by their family (HMG, 2014). However, forced marriage can also occur without HBA; for instance, it may be a route out of poverty, a means of securing care or protection for disabled relatives or widows (HMIC, 2015), or assisting claims for U.K. residence (HMG, 2014). For this study, forced marriage was conceptualized as one specific manifestation of HBA. Since HBA and forced marriage are often used interchangeably in the literature, work relating to both is reviewed.

HBA has been seen by some as primarily a “cultural” issue, associated with particular communities and arising from specific values around honor (Brandon & Hafez, 2008). The Iranian and Kurdish Women’s Rights Organisation has argued that it arises from “the culmination of an ideology of male dominance [and occurs when] the independence of [the] younger [generation] clashes with the cultural conservatism of elders who wish to maintain dominance” (Parliament, House of Commons, 2008, Ev 289–290). This has been seen as problematic, especially in terms of “othering” and scapegoating Black and Minority Ethnic (BME) communities. Okin (1999) has argued that seeing HBA as primarily “cultural” privileges culture over gender as a defining characteristic, thereby side-lining and minimizing the critical concept of unequal gender power relations.

In the United Kingdom, Black and South Asian feminists have argued against framing HBA as a problem of religion or “culture,” showing the dangers of “othering,” “essentializing,” and “racism” which can arise from so doing (Siddiqui, 2014; Thiara & Gill, 2010). Instead, many have interpreted it as one (of many) forms of violence against women (Begikhani et al., 2015; Payton, 2014).

Idriss (2017) has argued differently, that HBA should be seen as distinct in nature, due to the characteristics of collectivity, the organized nature of violence and the longevity of families’ desire for punishment. While these features of difference are generally recognized in theoretical and policy literature, Idriss argues that they should be given greater prominence, not just as points of difference, but sufficient to redefine HBA as substantively different to other forms of domestic violence.

Others (Dustin & Phillips, 2008; Sen, 2005) have shown that it is possible to adopt an approach which straddles arguments of similarity and difference. Just as Siddiqui (2014) warns of the dangers of collapsing all domestic violence against BME women into a category of HBA, thereby creating a “parallel universe”, Dustin and Phillips (2008) argue that, while HBA should be treated as “part of the wider category of domestic violence and violence against women,” it is important not to risk “blurring important differences” (p. 15). Sen (2005) and Payton (2014) demonstrate that HBA can be framed as a form of gendered violence without ignoring or minimizing the features of difference that exist, such as the greater involvement of women in planning or carrying out abuse, or the involvement of multiple perpetrators.

The approach adopted by successive governments in England and Wales has been to frame HBA as a form of violence against women, but to treat it as an issue requiring separate attention. While the cross-government definition does not explicitly identify HBA as gendered, the government responds within an overarching national strategy on gendered violence (HMG, 2016). Both government and national prosecution guidance identify that women are predominantly the victims, and that abuse is often used to assert male power to control female autonomy or sexuality (CPS, n.d.; HMG, 2014). Yet, governments and public authorities have also used law, action plans, and professional guidance to tackle specific forms of HBA (e.g., criminalizing FGM and forced marriage, statutory multiagency guidance on forced marriage).

### Who and What Are Involved

HBA and forced marriage are associated in the literature with more male victims and female perpetrators than other domestic abuse cases, yet remain heavily gendered. In England and Wales, national prosecution data show that women were 72% of victims and 14% of defendants in HBA cases (men were 28% and 86%, respectively) (CPS, 2017). The same data set for domestic abuse found women were 83% of victims and 8% of defendants (men were 17% and 92%, respectively).

Limited available figures nationally suggest victims of HBA are often younger women. This is particularly the case where there is a forced marriage, with victims often less than 25 years of age and many less than 18 years old (Foreign and Commonwealth Office [FCO], 2016; Hester et al., 2008; Kazimirski et al., 2009). In the U.K. context, identified cases of HBA often involve BME victims (and perpetrators), particularly from South Asian ethnicities. This likely reflects both the historically larger South Asian diaspora communities in this country, and also that feminist activism around HBA has been longer established within these communities. Victims have also been identified from a wide range of other ethnicities and communities, including Middle Eastern, Arab, African, and Eastern European (e.g., Hester et al., 2015; Karma Nirvana, 2008). It is associated more with Muslim communities nationally, but, as with ethnicity, this may be a reflection of a large South Asian Muslim population in the United Kingdom.

Immigrant spouses have been particularly identified with HBA, domestic abuse among BME communities and, to some extent, forced marriage (Hester et al., 2015). There do not appear to be any current data on what proportion of HBA victims may have vulnerable immigration status, although Dyer (2015) found that of the victims of “honor killings” identified in the United Kingdom over 5 years, none were British nationals. Perhaps in contrast, victims of forced marriage have been shown more often to be British citizens and/or to have secure immigration status (Hester et al., 2008; Kazimirski et al., 2009).

Certain key features are regularly highlighted as making HBA distinctive–in particular, collectivity of perpetration (multiple perpetrators, and/or evidence of premeditation or conspiracy), and the role of women in policing other women’s behavior and carrying out punishments (Aplin, 2017; HMG, 2014; Idriss, 2017; Payton, 2014; Sen, 2005; Begikhani et al., 2015). Views differ, however, as to how much these differences should be seen as variation between different culturally specific forms of violence against women, or whether they delineate HBA as inherently different.

In terms of the abusive behaviors, published data show that HBA can involve controlling behavior (e.g., removing victims from school or education, controlling freedom of movement, preventing the victim from learning English, threatening to deport immigrant spouses, and threats to remove children); physical abuse (including from family members and specific forms of attack such as acid attacks); financial abuse; sexual abuse (including around forced marriage); psychological and emotional abuse to the victim and sometimes to third parties (e.g., threats to harm family members, threats and humiliation to the victim, enforced servitude, and shaming to the community); victims being kidnapped or forced to travel abroad; and spousal abandonment (Brandon & Hafez, 2008). Forced marriage is commonly associated with HBA, with as many as 80% reporting threats of a forced marriage, or one having taken place (Karma Nirvana, 2008).

### Why Relationships Are Important

Hester (2013) has established the importance of victim–perpetrator relationships in understanding and developing responses to domestic and interpersonal abuse. While HBA is often associated with multiple perpetrators and family/community collusion (e.g., Aplin, 2017; Idriss, 2017), studies have shown that victim–perpetrator relationships vary. Cases often include the victim’s male (patrilineal) blood relatives or in-laws: their father, brother(s), cousins, and/or uncle(s) (Payton, 2014).

Other evidence has highlighted the involvement of intimate partners—with or without others (e.g., Karma Nirvana, 2008). Dyer (2015) found that over half of “honor killings” in the United Kingdom involved a current or former partner and/or that partner’s family; the rest involved the victim’s parents. The National Police Chiefs’ Council recognize that “much of the abuse does originate from intimate partners and the immediate family, although further abuse can be instigated by extended family members or members of the community” (National Police Chiefs’ Council, 2015, p. 15).

So, relationships are key to understanding who does what to whom. While much attention has been focused on the existence of multiple perpetrators and the extended family involvement—features of difference from domestic abuse, less light has been shone on the involvement of intimate partners—features of similarity.

## Method

### Case File Analysis

Prior research on gender-based violence has used case file analysis of criminal justice data to examine the nature of rape, domestic violence, and murder, and to develop case typologies (e.g., Dobash & Dobash, 2015). Using existing case records has several benefits, including accessing rich sources of unexplored data, avoiding some of the sampling challenges with a hard-to-reach population (e.g., how to identify participants and sampling biases in self-selection of participants), overcoming comprehension issues in communities for whom English is not a first language, and ethical benefits such as not retraumatizing victims (Hayes & Devaney, 2004). Permission was granted for the research by a University Research Ethics Committee in Southern England.

### Data Sample

Since 2018, police forces in England and Wales have flagged police incident reports as HBA. Flagging involves the initial responding or reviewing officer in a case applying an electronic marker to any incident or crime record if they believe HBA to be involved. This flag then follows the case through to closure—whether that is just an initial safeguarding check, or the case goes through to investigation and prosecution. Other than internal police force monitoring, prior research did not seem to have utilized these flagged cases (Aplin, 2017, has since published work on police HBA-flagged cases).

Access was granted to police records of incidents and/or crimes flagged as HBA in one police force in Southern England. The force was sampled opportunistically, being the only one of seven initially approached where research access was granted within the timeframe required. The police cases were supplemented by case records flagged as HBA by caseworkers in two specialist support services for HBA victims, one in Northern England and one in the East Midlands of England. These services were selected because they were in different parts of the country to the police force (i.e., to sample more areas), and because they reported sizable numbers of HBA cases. In these services, flagging followed a similar process as that in the police: if, in the judgment of the caseworker assigned to support a victim, the situation involved a risk of HBA, they applied a marker to the electronic case file to indicate this. All the cases sampled were tracked from report to that agency to the point the agency closed the case. Records included demographic data on victims, perpetrators, the nature of the abuse, and the progression of the case. At all three sites, 100% of cases opened in a 12- to 15-month period from the end of March 2014 were sampled, as the most recent available set of cases. Data were extracted and coded by the researcher from the database at each site, duplicates and partial records removed, and the remaining cases anonymized on-site. Anonymized data were transferred to the university’s secure server via encrypted memory stick, as per data agreements signed with each site. Combined, a total of 162 cases were extracted, and these are referred to as the “162-case data set.”

To test emerging findings from the 162-case data set using quantitative analysis, a bigger data set was needed. Through professional contacts, access was secured to a precoded and anonymized quantitative data set of 1,312 cases of HBA collected from caseworkers in local domestic abuse services by the national charity SafeLives. These cases were originally flagged as HBA in the same process described above for the victims’ services—i.e., if the caseworker judged the incident to involve a risk of HBA, they applied an electronic marker to the case record. These cases were opened between 2010 and 2015. The data were transferred in .xml file via encrypted memory stick. This is referred to as the “Insights data set.”

A harmonized set of variables was developed consisting of victim, perpetrator, and abuse profile data across all 1,474 cases. In addition, a short descriptive case summary was drawn up for each of the 162 case records, giving a brief overview of who was involved, what the abuse was and any context or triggers for it (e.g., use of terms to do with honor/shame), what actions the police/victim service had taken, and what the situation was at case closure. For the police force, these summaries already existed in the police database as incident summaries; for the victim services, they were created from the case files.

### Analysis

To examine victim–perpetrator relationships, each case in the 162-case data set was coded for all the different perpetrators involved. Initially, this comprised 11 codes (e.g., “husband + sisters-in-law” or “own father + uncles + cousins”). These were re-coded in several rounds using grounded methods, revisiting the case records until the categories were saturated and then collapsing similar categories together (Glaser & Strauss, 1967). The final three groups were:

1. Abuse from a (current or ex) intimate partner only (which I call “Type 1: partner only”). Occasionally these cases involved explicit or implicit pressure (but no direct violence or abuse) from other family members, usually on the victim to remain in the relationship–for example, communications from in-laws and/or extended family members encouraging the victim not to divorce and saying it would be harmful to the family reputation.
2. Abuse from family members only (which I call “Type 2: family only”). No intimate partner was involved; abuse was from one or more natal family members or in-laws.
3. Abuse from both intimate partner and family members (which I call “Type 3: partner plus family”). A current or ex intimate partner and one or more natal family members or in-laws.

These three types were replicated in the Insights data set using a proxy variable.^2^ Descriptive statistics were run on the combined data sets to test for significant associations between victim, perpetrator, and abuse profiles and the three types. Standardized residuals for the Pearson’s chi-square test for each variable indicated which of the three types held the association.

Characteristics found to be significantly associated with the types were further tested using multinomial logistic regression in the Statistical Package for the Social Sciences (SPSS) on the Insights data set (the only data set large enough to be valid for three-way modeling on multiple variables) to see whether the associations held when other variables were controlled. Of the 10 variables in the Insights data set found in chi-square tests to be significantly associated with *type* (Table 1), *perpetrator gender* was excluded because it had over 50% missing data; *multiple abuses* were excluded because it was double counting the other abuse variables; and *physical abuse* was removed from the model as it became nonsignificant when entered with other variables (indicating a potential link with another predictor variable).

**Table 1.** Pearson’s Chi-Square Associations of Victim, Perpetrator, and Abuse Variables With Type.

Variable	Categories	162-case data set	Insights data set
Victim age (grouped)	<25 years	28.914***	224.370***
	25+ years		
Victim gender	Includes female	3.945	9.437**
	Includes male		
Victim ethnicity (grouped)	South Asian	4.095	87.657***
	Non-South Asian		
Victim sexual orientation	Heterosexual	Test not valid	Test not valid
	LGBT		
Victim immigration status	No Recourse to Public Funds	3.366	39.058***
	Has Recourse/Don’t know		
Victim religion	Atheist	Test not valid	Variable doesn’t exist
	Christian		
	Hindu		
	Muslim		
	Sikh		
Multiple perpetrators	Single	Test not valid	Test not valid
	Multiple		
Primary perpetrator gender	Primary perp male	Test not valid	75.882***
	Primary perp female		
Includes a female perpetrator	Yes	50.714 (2) ***	Variable doesn’t exist
	No		
Jealous/controlling behavior	Yes	Test not valid	4.752
	No/Don’t Know		
Harassment/stalking	Yes	1.191	2.018
	No/Don’t Know		
Physical abuse	Yes	6.087*	30.287***
	No/Don’t Know		
Sexual abuse	Yes	21.982***	116.087***
	No/Don’t Know		
Threats to kill	Yes	4.846	Variable doesn’t exist
	No/Don’t Know		
Forced marriage	Yes	30.039***	195.214***
	No/Don’t Know		
Risk	Non-High risk (<10)	Variable doesn’t exist	38.723***
	High risk (10+)		
MARAC threshold	Yes	Variable doesn’t exist	1.267
	No		
Multiple abuses	One form	10.060	35.867***
	Two forms		
	Three forms		
	Four forms		
	Five forms		
	Six forms		

*Note.* LGBT = lesbian, gay, bisexual, or transgender; MARAC = multiagency risk assessment conference.

* Significant at *p* < .05. **Significant at *p* < .01. ***Significant at *p* < .001.

The final model (Table 2) contained 1,132 valid cases. The model χ^2^ (488.158) was significant (*p* < .001), showing that the model was significantly better at predicting whether or not a case would fall into *Type 2* or *Type 3* (rather than *Type 1*) compared with the base model with no predictor variables included. Therefore, the null hypothesis (that the inclusion of predictor variables makes no difference to the predictive power of the model) was rejected. A Nagelkerke *R*^2^ value of .395 showed that the model explained 39.5% of unexplained variance in the data. Checks were run for multicollinearity; none was found.

**Table 2.** Multinomial Logistic Regression, Predicting Variables to Type.

	Final model (*n* = 1,132)			
Types 2 and 3 compared with Type 1 (Ref.)				
	*B* (*SE*)	Wald	Exp (*B*)	95% CI [low, high]
Coefficients				
Intercept (Type 2)	−1.022 (0.353)	8.396	—	—
Intercept (Type 3)	1.252 (0.259)	23.400	—	—
Victim age				
Less than 25 years (Type 2)	2.004 (0.213)	88.431***	7.421	[4.887, 11.269]
Less than 25 years (Type 3)	0.254 (0.219)	1.343	1.289	[0.839, 1.981]
25 years or above (Ref)	—	—	—	—
Victim gender				
Male (Type 2)	1.874 (0.540)	12.033**	6.517	[2.260, 18.791]
Male (Type 3)	0.908 (0.548)	2.742	2.479	[0.846, 7.260]
Female (Ref)	—	—	—	—
Victim ethnicity				
Not South Asian (Type 2)	−1.191 (0.193)	37.943***	0.304	[0.208, 0.444]
Not South Asian (Type 3)	−0.945 (0.155)	36.981***	0.388	[0.286, 0.527]
South Asian (Ref)	—	—	—	—
Victim immigration status				
Recourse to public funds (Type 2)	0.313 (0.240)	1.707	1.367	[0.855, 2.187]
Recourse to public funds (Type 3)	0.476 (0.172)	7.639**	0.621	[0.443, 0.871]
No recourse to public funds (Ref)	—	—	—	—
Sexual abuse				
No (Type 2)	1.475 (0.258)	32.689***	4.369	[2.636, 7.243]
No (Type 3)	−0.511 (0.157)	10.634**	0.600	[0.441, 0.815]
Yes (Ref)	—	—	—	—
Forced marriage				
No (Type 2)	−1.720 (0.202)	72.342***	0.179	[0.120, 0.266]
No (Type 3)	−0.525 (0.200)	6.860**	0.592	[0.399, 0.876]
Yes (Ref)	—	—	—	—
Risk				
Non-High risk (under 10) (Type 2)	0.430 (0.186)	5.353*	1.537	[1.068, 2.213]
Non-High risk (under 10) (Type 3)	−0.302 (0.168)	3.232	0.740	[0.532, 1.028]
High risk (10+) (Ref)	—	—	—	—
Model χ^2^ (*df*)	488.158 (14)***			
−2LL	377.299 (Intercept only model: 865.457)			
Nagelkerke *R*^2^	.395			

* Significant at *p* < .05. **Significant at *p* < .01. ***Significant at *p* < .001.

## Findings

### Victim–Perpetrator Relationship: Three Types

To answer the first research question, “Who does what to whom?,” the victim–perpetrator relationships were examined. The majority (71%) involved a current or ex intimate partner perpetrator. Where an intimate partner was not involved, perpetrators were the victim’s natal family or (less commonly) their in-laws. The cases were divided into three types, based on the combinations of victim–perpetrator relationship: victims abused only by an intimate partner (Type 1) accounted for 40% of cases; those abused by one or more family members (Type 2) for 29%; and those abused by both (Type 3), 31%. Table 1 (Pearson’s chi-square associations of victim, perpetrator, and abuse variables by type) shows the results of statistical tests for association between the three types and case characteristics.

### Victim Characteristics (“To Whom”)

#### Victim age

Type 2 victims were younger, with the majority of victims aged 24 years and younger, compared with Types 1 and 3 where the majority of victims were above 25 years (most were between ages 25 and 44 years). This age split at 25 years between Type 2 and the other types was statistically significant (Table 1).

#### Victim gender

All types were strongly gendered, with more than 88% in each type including a female victim, compared with less than 20% a male victim. Although still small in number, Type 2 was significantly more likely to involve male victims (Table 1).

#### Victim ethnicity

While victims across all types were most likely to be South Asian, this ethnicity was significantly more likely in Types 2 and 3 (around three quarters of cases), whereas White victims were significantly associated with Type 1 (Table 1). Similar (smaller) numbers of cases across all types were of Black African/Caribbean and Middle Eastern/Arab ethnicities.

#### Victim sexuality

More than 90% were in heterosexual relationships. Type 2 had very slightly higher proportions of Lesbian, Gay, Bisexual, or Transgender (LGBT) victims, but only by one or two cases; and there were too few LGBT victims to run statistical tests.

#### Victim immigration status

Type 3 victims were significantly more likely to have No Recourse to Public Funds (NRPF). This suggests Type 3 involved more immigration-vulnerable immigrant spouses (living with husbands and in-laws), whereas Types 1 and 2 involved more British women.

#### Victim religion

Where known, victims were most commonly Muslim. However, religion was unknown in the majority of cases. Victims were also Hindu, Christian, and Sikh. There was too much missing data to observe or test differences between types.

### Perpetrator Characteristics (“Who Does”)

Overall, primary perpetrators were men across all types: at least 72% where gender was known. Primary female perpetrators, though few, were slightly more common in Type 2 compared with the other types, and this link was statistically significant (Table 1). The involvement of a secondary female perpetrator (i.e., as well as a primary male perpetrator) was high in Type 2 (65%) and Type 3 (67%) compared with Type 1 (7%), also statistically significant (Table 1). Closer examination of these cases suggested that female perpetrators tended to be the victim’s own mother in Type 2, whereas in Type 3 it was most commonly their in-laws (mother-in-law and/or sisters-in-law).

### Abuse Characteristics (“What”)

#### Forms of abuse

As with overall domestic abuse (Safelives, 2015), across all types, jealous and controlling behavior was the most common abuse, in more than 80% of cases. Harassment and stalking were similar across the types. The rate of threats to kill was notably higher in Type 3 (58%) compared with Types 1 and 2 (30% and 34%); however, Types 1 and 2 had more missing data for this variable. Although the proportions of these three forms of abuse varied slightly across the types, they were not statistically significantly more likely in one or another. By contrast, the abuse forms which were significantly associated with type were physical abuse, sexual abuse, and forced marriage. Physical abuse was significantly more common in Type 3 and least in Type 2 (Table 1). As might be expected, sexual abuse was significantly associated with Types 1 and 3, which involved intimate partner perpetrators (Table 1). Forced marriage was significantly associated with Type 2, occurring in over half of those cases compared with around one fifth of the other types (Table 1).

#### Risk

Type 3 was most likely to be scored high risk, and Type 2 least likely, using the domestic violence risk assessment tool “Domestic Abuse, Stalking and Honor-Based Violence (DASH).” DASH is commonly used by victim services and police in the United Kingdom, and consists of a checklist of 14 questions that identify risk factors, plus the professional judgment of the practitioner making the assessment (Safelives, 2019). This link of risk to type was significant (Table 1). However, all three types were equally likely to meet the risk threshold for the case to be referred to a domestic violence multiagency risk assessment conference (MARAC). This may suggest that Type 2 cases score lower on the actuarial risk tool (DASH), though are escalated to MARAC for other reasons (e.g., professional judgment related to perceived risk from honor context).

#### Multiplicity of abuse

Less than one third of all cases involved just one form of abuse. This was particularly pronounced for Type 3 victims, where more than 85% experienced multiple. The association of multiple abuses with Type 3 was statistically significant (Table 1).

### Logistic Regression

The victim, perpetrator, and abuse characteristics found to be significantly associated with the types were tested using multinomial logistic regression to see whether the associations held when other variables were controlled.

*Primary perpetrator gender, physical abuse*, and *multiple abuses* all became nonsignificant when other variables were controlled for. All seven remaining variables—*victim age, victim gender, victim ethnicity, victim immigration status, sexual abuse, forced marriage*, and *risk*—were confirmed to significantly predict whether a case would fall into Type 2 or 3 (compared with Type 1), when holding other variables steady (Table 2). For each of these seven variables, regression analysis confirmed the relationship effects observed from standardized residuals in the descriptive statistics. For two variables, an additional relationship was shown. *Sexual abuse* was confirmed to be significantly least likely in Type 2, but it was also significantly more likely in Type 3 than Type 1. This is interesting because both Types 1 and 3 involve intimate partner perpetrators, but this suggests Type 3 is more likely to involve sexual violence. *Forced marriage* was confirmed to be most strongly associated with Type 2, but regression showed it also to be significantly more likely in Type 3 than Type 1. This is interesting because most Type 3 victims were already married; thus, it suggests a sizable proportion of these considered their marriage to have been forced.

### Summary: Key Features of Types

Taken together, the results of the descriptive statistics and regression analysis show that Type 2 was the most distinct: these cases were more likely to involve younger victims (aged less than 25 years), male victims, and (together with Type 3) South Asian ethnicity victims. Type 2 was more likely to involve natal family members. Two thirds involved a female perpetrator (usually in a secondary role, although 20% in a primary role), more than the other types. Female perpetrators were most commonly the victim’s own mother. Type 2 cases were most likely to involve (threatened or actual) forced marriage, and less physical abuse. They were rated lower on actuarial risk tools but were as likely to be referred to high-risk multiagency case conferences (MARAC).

With regard to Type 1, partner abuse was significantly more likely to involve a White victim, although as with all the types, the majority of cases in this sample involved South Asian victims. In common with Type 3, most victims were aged between 25 and 44 years and were female. Most involved a single intimate partner perpetrator, and very few of these cases involved a primary or a secondary female perpetrator. Type 1 was most likely to involve physical abuse and, together with Type 3, was more likely than Type 2 to involve sexual abuse.

In Type 3, partner plus family abuse, shared characteristics with Type 1 (e.g., abuse profile, risk level, and involvement of intimate partner), but these characteristics were amplified in almost every way—for example, Type 3 victims had more threats to kill, the highest number of different forms of abuse, and was significantly most likely to involve sexual abuse. Type 3, however, was distinct from Type 1 in two key ways: first, the involvement of multiple family member perpetrators (generally in-laws, and often a mother-in-law) in addition to an intimate partner; second, the high proportion of immigration-vulnerable victims (those with NRPF).

## Discussion

This article sets out to answer two questions to address a gap in knowledge about the nature of HBA in England and Wales. The previous findings section answered the first question (who does what to whom?) by analyzing data on the victims, perpetrators, and abuse. It proposed the existence—at least in the context of cases known to police and victims’ agencies in England and Wales—of three types of HBA, defined by the relationship between victim and perpetrator(s). While the involvement of intimate partner perpetrators in HBA is identified in the literature (Dyer, 2015; Karma Nirvana, 2008), most policies and definitions, as well as much of the literature, strongly emphasize family member perpetrators and collectivity (Brandon & Hafez, 2008; HMIC, 2015; Payton, 2014). Indeed, involvement of multiple family and community members is often cited as a distinct factor. A key finding of this study is the evidence it provides of the strong involvement of intimate partners—both alone (Type 1) and with others (Type 3)—in cases identified by professionals as HBA.

This discussion section addresses the second question: How is HBA similar to or different from other domestic and intimate partner abuse? It draws conclusions from the profile of cases across the whole combined data set, and from comparing the three proposed types of HBA. Finally, implications for policy are suggested.

### Is HBA Similar to or Different from Other Domestic Abuse?

First, these findings show that HBA (like other domestic abuse) is primarily gendered. Second, the cases have specific features in common with domestic and intimate partner abuse, which suggests that some violence against BME women has been artificially separated from mainstream domestic abuse. Third, there are cases with specific features of difference, which may argue for their separate treatment. The key to teasing out these differences is the three types of case I propose, which can be arranged along a spectrum of commonality to difference.

#### HBA is gendered—but cultural tools apply

This study found strong evidence, first, that HBA is gendered in terms of victimization and perpetration, with 94% of cases involving a female victim, and 92% of the primary perpetrators were male (where gender was known). This is a higher female victim rate than reported before in official data (FCO, 2016; HMIC, 2015), though recent research using police data found a similar rate (Aplin, 2017). Second, these findings support arguments that the primary defining feature of HBA is gender inequality and patriarchal values which privilege control of women’s autonomy and sexuality.

At the same time, I suggest these cases do show that cultural values and practices, in the form of ideas of honor and shame invoked by the perpetrator or wider family members, also play an important part. These cultural practices are second in importance to gender, but nevertheless identifiable. I would question, however, whether the cultural elements in HBA cases may just be more “visible” to Western eyes, when in fact honor can be viewed as one of many cultural tools used by abusers—at least when it is abuse from an intimate partner. Within Type 1, there was a subgroup where the intimate partner explicitly used honor as a weapon of control or intimidation. While this looked different on the surface, it would be possible to replace the use of honor with other tools of control used by domestic abuse perpetrators. In the same way that perpetrators were using honor/shame to threaten, cajole, intimidate, shame, or plead with their victim, other abusers might use children, financial control, sexual abuse, or threats to shame the victim with sexual information or images, and so on. So, at least in Type 1, we can observe that honor is used as a culturally specific weapon of control but, critically, it could be replaced with other “culturally specific” tools in cases of White British domestic abuse. This perhaps underlines how practices in BME communities are often labeled “cultural,” whereas those specific to mainstream (White) cultures are labeled “gendered” (i.e., culture is ignored; Chantler & Gangoli, 2011).

#### Commonalities with domestic and intimate partner abuse

In addition to their gendered nature, the key feature that many of these HBA cases shared with domestic intimate partner abuse was the fact that perpetrators were an intimate partner. Other common elements were that most victims were in their 20s and 30s; the most common form of abuse was jealous or controlling behavior; there was often physical abuse, harassment, and/or stalking, and (less often) sexual abuse; there were often multiple forms of abuse and much of it was deemed high risk.

The existence of Type 1 cases demonstrates that a sizable portion of what is being identified by police and victims’ specialist services as HBA looks much like intimate partner abuse (i.e., what would be called domestic violence in a White British family). This supports Siddiqui’s (2014) “parallel universe” argument that different forms of violence against BME women have been collapsed through the lens of honor-based violence, or Brandon and Hafez’s (2008) “honor-based domestic abuse” in which domestic abuse in BME communities is branded as HBA. One risk of seeing all BME domestic abuse as honor-related is that it oversimplifies BME women’s experiences, leading to their “collective victimhood” and disguising very different experiences (Thiara & Gill, 2010). These case data suggest that BME women experience three different types of abuse, all called HBA, but with quite varied characteristics. That a mediating context of “honor” often operates implicitly (and therefore perhaps invisibly) should not, of course, be taken as evidence that it does not exist, or is not powerful. There is previous research evidence to show that victims of intimate partner abuse frequently report that “honor” mediates and magnifies their experiences of abuse (Hester et al., 2015).

#### Specific, distinct elements?

Across all these HBA cases, there were proportionally more male victims than other domestic abuse cases, cases with BME victims (principally South Asian), non-British national victims, victims with insecure immigration status, cases with multiple perpetrators, and cases involving a female perpetrator. While the profile of abusive behaviors was similar to other domestic abuse, more cases involved a forced marriage, and sizable numbers involved threats to kill.

These distinct elements were more pronounced in Type 2 (and to a lesser extent, Type 3). Type 2 differed the most in that it did not involve intimate partner violence, was more likely to involve forced marriage, and to involve abuse from the victim’s own family members, generally relating to their rejection of the family’s preferred marriage, or the family’s rejection of the victim’s choice of partner, or occasionally their lifestyle choices (e.g., education and western dress). It also more often included female perpetrators, especially mothers. The profile of Type 2 finds support in existing literature on forced marriage which identifies victims as being predominantly British nationals, but the purpose of the intended marriages being commonly to maintain commitments to family or communities overseas (HMG, 2014; Kazimirski et al., 2009), and of these types of cases involving abuse and coercion from the victim’s family members relating to a young person’s marriage or sexuality preferences (Hester et al., 2008).

Type 3 also contained specific elements distinguishing it from mainstream domestic abuse: the involvement of female perpetrators (most commonly mothers-in-law and other female in-laws), domestic servitude, and victims with insecure immigration status. Again, this profile is supported by existing literature which identifies a group of immigrant wives on spousal visas who are particularly vulnerable to experiencing domestic abuse from their partner and extended family, often involving extreme domestic servitude and isolation (Siddiqui, 2014).

Both Types 2 and 3 had the distinctive feature of involvement of one or more female perpetrators. While the involvement of female perpetrators in these cases seems higher than in other forms of domestic abuse, they seem to play varying roles—for instance, Type 2 involves more mothers but there is a question mark about whether they are actively involved or may be acting as mediators or even protective figures; whereas Type 3 involves more mothers-in-law and sisters-in-law, seemingly in more proactive roles, which fits with the literature on mother-in-law to daughter-in-law violence (e.g., Payton, 2014) and the hierarchical structures of some South Asian extended families—the “culturally specific form of patriarchal bargain” identified by Rew et al. (2013). The roles of these women in HBA—which are themselves complex and must not be collapsed or “essentialized”—can still be seen as part of the gendered nature of this abuse when seen through the lens of the patriarchal bargain.

#### A continuum?

I suggest that the three types can be laid out on a continuum from similarity to difference, in the order 1–3–2. Type 1 can be seen as pretty well synonymous with “mainstream” domestic and intimate partner abuse, apart from the ethnicity of the victim or perpetrators. It always involved a single perpetrator who was the current or ex intimate partner, with multiple forms of abuse often involving jealous and controlling behavior and physical abuse, much of which was high risk. A subgroup of Type 1 had the same profile but with a background context of control or intimidation relating to pressure from honor/shame; this was either the intimate partner themselves using honor/shame as a cultural tool of control, or pressure from one or both families (but no direct abuse), generally for the victim and perpetrator to stay together.

Type 3 shared many elements with Type 1, abuse from a current or ex intimate partner, but with additional perpetrators, who were most often the victim’s in-laws. The profile of the victims differed in that they were more likely to be immigrant spouses, with insecure immigration status, experiencing more forms of abuse and deemed to be higher risk. Type 3 could be characterized as an extension of intimate partner domestic abuse, where in-laws join partners in abusing and controlling the victim.

Most different, and with the most specific features, was Type 2, involving abuse from family members only, usually natal family members, and with a distinct profile of victims (mainly female, but more male), perpetrators (mainly men but many more women involved), abuse, and risk, and most likely to involve forced marriage.

### What Does This Mean for Conceptualizing HBA?

On the basis of these findings, I suggest that Type 1 should be seen as the same as “mainstream” domestic intimate partner abuse, with a recognition that there is sometimes a mediating influence of honor and related pressure from extended family members (Hester et al., 2015). Type 3 should be seen as a specific form of BME domestic intimate partner abuse, with its own risks and particularities stemming from the involvement of multiple extended family members alongside the partner, and recognizing the distinct profile features of victims in this group. This reflects a pattern of domestic abuse particular to BME families already identified by Siddiqui (2014). It is Type 2 that is most different from the others in terms of characteristics relating to the victim, perpetrator, and nature of the abuse, and the only one that cannot be construed as domestic intimate partner abuse. While it does fit the wider definition of (familial) domestic abuse, it has the greatest differences in terms of victim and abuse features, risks, and the range and roles of perpetrators. Thus, Type 2 could be framed as a distinct form of abuse separate from the other types, and offers support for those who argue in favor of defining (some) HBA as substantively different from other domestic abuse (e.g., Idriss, 2017). In sum, all three types can be seen as forms of domestic abuse in which “honor” mediates abuse from intimate partners and/or family members. At the same time, *some* HBA cases can be seen as significantly different from intimate partner abuse, while others can be seen as particular forms of intimate partner abuse.

### Limitations

The first key limitation of this study is the possibility that the three types are reflective only of the flagging practices of particular agencies, in particular if police or victim agencies are identifying as honor-based cases that are really “just” domestic abuse, probably based on the ethnicity of the victim and/or perpetrator. While HMIC (2015) found that most police forces did flag incidents as HBA, the inspection also found that the consistency of flagging varied considerably between forces and expressed concern that if officers did not understand HBA they would not correctly identify and flag it. This study has contributed to the debate on flagging, by showing how flagged cases can be examined and compared through research, to improve definitions and understandings of HBA. But it is also possible that the cases sampled in this study are limited by the way in which the police and victims’ agencies interpreted and applied the HBA flags.

The second key limitation is that the types identified may be a particularity of the cases sampled—reflecting perhaps the demographic profile of the area or types of agency from which data were extracted, or the willingness of victims to report to particular agencies. For instance, individuals from BME communities may be mistrustful of the police and less likely to report abuse to them, or engage with police processes (End Violence Against Women Coalition, 2019). Thus, while the police cases only account for a minority of the total sample in this study, it is possible that police files capture specific groups of victims.

This is only an exploratory study and, as such, does not make strong claims about generalizability. However, while both these limitations which relate to sample biases are possible, the fact that the types and profiles of cases were replicated in data from different agencies—victims’ services as well as police–—and from 48 different domestic abuse services across England and Wales, suggests that the findings do not just reflect the culture or practices of a particular agency or geographical demographics. It would be fruitful to test and replicate the typology in other agencies and areas; in particular, to examine whether the proportion of cases of each “type” varied by agency, area of the country, and local population demographics.

### Policy and Practice Implications

#### Policy definitions

What might these types mean for policy definitions? In terms of domestic abuse, all the cases fit the government definition: that is, abuse from an intimate partner or family member. In addition, many of the cases (especially Types 1 and 3) also fit a narrower definition of intimate partner abuse. In terms of definitions of HBA, the picture is more complex. These findings show the U.K. Government’s definition of HBA not to be wrong, but to be inadequate on several grounds. First, that definition states HBA is “an incident or crime.” This study has shown that (as with domestic abuse and reflected in the related offenses of stalking and harassment, and coercive and controlling behavior), HBA should rather be seen as a *pattern* of incidents and acts, and rarely as a *one-off incident*. Second, it states that abuse is committed “to protect or defend the honour of the family and/or community.” This again only captures a partial picture. This article has shown that honor is involved in more nuanced ways than always as a direct trigger for violence; for instance, in Type 1, honor may simply represent an implicit or hinted-at context mediator in domestic abuse cases. Reference to the family and/or community points to multiple perpetrators and also misses the element of personal honor, and individual perpetration, which Type 1 cases in particular display. Third, the definition omits any mention of who or what is involved in HBA, and of the gendered nature of abuse.

So, the existing Government definitions of domestic abuse (including intimate partner violence) and HBA do fit, but only describe part of the picture. Drawing on the empirical findings in this study, I propose a revised definition of HBA, which makes the people involved more visible, indicates the direction of abuse between them, highlights the involvement of intimate partners, and emphasizes the gendered nature of HBA, situating it as a pattern of behavior with links to domestic and intimate partner abuse:

Honor-based abuse is any incident, or pattern of incidents, of controlling, coercive, intimidating, or threatening behavior or abuse (which may include psychological, emotional, physical or sexual abuse, isolation, abandonment, forcing someone to marry, threats to kill, murder, kidnap, or other acts of domestic abuse) carried out by one or more family members and/or a (current or former) intimate partner, to protect or defend the honor of an individual, family and/or community against perceived or anticipated breaches of their code of behavior, regardless of the age, ethnicity, sexual orientation, religion, or gender of the victim. It is a form of (primarily male) violence towards (primarily) women.

#### Consequences for policy and practice

These three types of HBA have implications for how victims’ nongovernmental organizations (NGOs), statutory services, and government respond to HBA. The characteristics identified here to be associated with each type could help practitioners identify both risk and protective factors and improve victims’ safety.

Type 1 suggests that frontline practitioners, especially police, need to review their definitions and understandings of HBA; for instance, are all officers trained to recognize HBA and is there sufficient guidance and training on what counts as HBA and what might be “just” domestic abuse in a couple where one or both partners are BME? Police forces could review a sample of their cases flagged as HBA to see whether officers are consistently and correctly identifying it according to the definitions they have adopted. Training for police and other practitioners (social workers and teachers), and central government guidance on HBA, should highlight that, even in cases that are “just” domestic intimate partner abuse, where one or both partners come from an honor culture, there may be particular pressure from in-laws and extended family members related to separation, divorce, and child custody.

Type 2 is the profile of case which most closely matches what many victims’ NGOs and police define as HBA. Here, the findings show that policy and practice guidance for police, social workers, teachers, and NGOs should recognize that in cases with younger victims (especially less than 25 years) and/or a risk of forced marriage, it is more likely that the victim’s mother may be involved in carrying out abuse. The dividing line of 25 years is new; often, forced marriage is seen to relate to young people between 15 and 18 years, but these data show that group extending up to 25 years. As well, police and victims’ NGOs should be aware that these Type 2 cases may score less highly on the DASH risk assessment tool, but the case may still be very risky.

With Type 3, victims are typically older (20s, 30s, 40s) and more likely to have vulnerable immigration status. This profile of victim is likely to be more hidden from police and other statutory services, due to the nature of domestic servitude in which many are kept, and barriers of language and fear about deportation. The existence of this group as a significant portion of HBA cases in England and Wales ought to be explicitly included in government and police practice guidance and training, and in local practice (e.g., local authority definitions of HBA). Immigration agencies and officials also should be trained to understand that this group are often victims of HBA. The government should consider whether current domestic abuse exemptions under immigration rules (e.g., the Domestic Violence Concession) could be amended to better recognize this group of victims. Type 3 is characterized by abuse from multiple people, often in-laws and mothers-in-law; and this group was most likely to experience multiple forms of victimization. Both these risk factors should be explicitly recognized in training for police, social workers, and immigration officials on HBA.

## Conclusion

Through quantitative analysis of a new, empirical data set of 1,474 cases of HBA identified by, and collected from, police and specialist victims’ agencies in England and Wales, this study has identified and defined three types of HBA, based on victim–perpetrator relationships. The cases examined show that the common (public and professional) perception in England and Wales of HBA as primarily parental abuse toward daughters (and sometimes sons) does not fit all cases. Instead, these new data show that HBA is frequently identified as taking place within intimate partner relationships, with or without the involvement of other family members. Much of the abuse profile is similar to other domestic and intimate partner abuse cases, in particular experiences of jealous and controlling behaviors, emotional, physical, and sexual abuse. And, as with domestic abuse, HBA is gendered, with the majority of cases involving a primary male perpetrator and a primary female victim, and with abuse frequently driven by patriarchal ideas about gender roles and the control of female sexuality or autonomy.

While further research is needed to test these findings with more HBA cases known to other agencies, the evidence presented in this article calls for a rebalancing of emphasis on the similarity of HBA to other forms of violence against women and intimate partner abuse, rather than any further divergence in policy, law, or definitions.

## Supplemental Material

VAW_ELP_updated_Bates – Supplemental material for Honor-Based Abuse in England and Wales: Who Does What to Whom? Click here for additional data file. Supplemental material, VAW_ELP_updated_Bates for Honor-Based Abuse in England and Wales: Who Does What to Whom? by Lis Bates in Violence Against Women

## Notes

1 While criminal justice agencies in England and Wales use the term “honor-based violence,” the term “honor-based *abuse*” is used in this article to resist a predominant focus on physical violence and instead recognize the breadth of victims’ experiences. Since 2015, “honor-based abuse” is also the term used by the National Police Chiefs’ Council in England and Wales.

2 A proxy variable was created, since the Insights data set did not record all perpetrators in a case, but just (a) the identity of the primary perpetrator, and (b) whether there were multiple perpetrators. Therefore, cases with a single, intimate partner perpetrator were assigned to Type 1; those with an intimate partner perpetrator but multiple perpetrators to Type 2; and those with family member perpetrators (whether single or multiple) to Type 3.
