# Supplementary material for: Honor-Based Abuse in England and Wales: Who Does What to Whom?
Source: Violence Against Women. 2020 Aug 31;27(10):1774–95. doi: 10.1177/1077801220952168 (PMC8239979; doi:10.1177/1077801220952168)
Supplement: VAW_ELP_updated_Bates – Supplemental material for Honor-Based Abuse in England and Wales: Who Does What to Whom? [file VAW_ELP_updated_Bates.pdf]

JOURNAL CONTRIBUTOR PUBLISHING AGREEMENT

For VIOLENCE AGAINST WOMEN (the "Journal")
Owned by SAGE Publications, Inc.
Published by SAGE Publications, Inc., 2455 Teller Road, Thousand Oaks, CA 91320 ("SAGE")

TITLE OF CONTRIBUTION (the "Contribution"): Honour-Based Abuse in England and Wales: Who Does What To Whom?

CORRESPONDING CONTRIBUTOR: Lis Bates
"Corresponding Contributor" refers to the lead author.

CONTACT INFORMATION: lis.bates@open.ac.uk +44 7843 046092

____

ALL CO-AUTHORS: ______________________________

____

As used herein, the lead author and all co-authors of the Contribution are collectively referred to as "Contributors" and individually as a "Contributor."

Please read the attached terms, then complete, sign and return this form by mail, courier, or email to the Editorial Office at

Claire M. Renzetti, Department of Sociology, University of Kentucky, 1561 POT, Lexington, KY 40506-0027
Email: claire.renzetti@uky.edu

EXCLUSIVE LICENSE TO PUBLISH

Contributors represent and warrant that they own the copyright in the Contribution unless one of the following is checked:

☐ **Work made for hire for employer/Work done in the course of employment**—the Contribution was prepared by the following Contributor, ________________________________, at the request of such Contributor's employer and within the scope of the Contributor's employment, and the copyright in the Contribution is owned by the Contributor's employer. (Both Contributor **and** an authorized representative of the Contributor's employer must sign this Agreement.) Please name **Employer**: ______________________________

☐ **U.S. Government work**—Contributors are employees of the United States Government **and** prepared the Contribution as part of their official duties. Please name **Government Employer**: ______________________________

(Please also read Section 2 of the attached "Terms of Agreement").

In consideration for publication of the Contribution in the Journal, Contributors hereby grant to SAGE the exclusive right and license to reproduce, publish, republish, prepare all foreign language translations and other derivative works, distribute, sell, license, transfer, transmit, and publicly display copies of, and otherwise use, the Contribution, in whole or in part, alone or in compilations, in all formats and media and by any method, device, or process, and through any channels, now known or later conceived or developed; the exclusive right to license or otherwise authorize others to do all of the foregoing; and the right to assign and transfer the rights granted hereunder. To the extent that any right now or in the future existing under copyright is not specifically granted to SAGE by the terms of this Agreement, such right shall be deemed to have been granted hereunder.

In the event Contributors provide Supplemental Materials, as defined in Section 2 of the Terms of the Agreement, Contributors hereby grant to SAGE the non-exclusive right and license to reproduce, publish, republish, create derivative works, distribute, sell, license, transfer, transmit, and publicly display copies of, and otherwise use, the Supplemental Materials, in whole or in part, alone or in compilations, in all formats and media and by any method, device, or process, and through any channels, now known or later conceived or developed, for the full legal term of copyright and any renewals thereof, throughout the world in all languages and in all formats, and through any medium of communication now known or later conceived or developed, and the non-exclusive right to license or otherwise authorize others to do all of the foregoing, and the right to assign and transfer the rights granted hereunder.

By signing this Agreement, Contributors acknowledge their agreement to the preceding provisions **and** the terms and conditions stated in the attached "Terms of Agreement." Contributors represent and warrant that they have completed all information on the attached "Additional Contributor Information" accurately and thoroughly.

By signing this Agreement on behalf of all Contributors, Corresponding Contributor represents and warrants that he/she has received written permission from each Contributor to sign this Agreement on his or her behalf and to grant the exclusive license herein. Corresponding Contributor understands that he/she has the option of having each Contributor sign a separate copy of this Agreement by contacting the Editorial Office for a version of this Agreement to be signed by each Contributor and returned directly to the Editorial Office.

For Contributors:

Signed: ………………………………………………………………………..……. …………………………Date: 5 May 2020 …………………………….

For Employer (only required if **Work made for hire/done in the course of employment** box is checked)

Signed: ………………………………………………………………………..……. …………………………Date: …………………………….
Authorized Representative: Name, Title:

For SAGE Publications, Inc.:

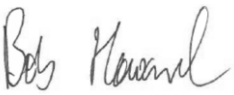

Bob Howard, Vice President, Journals

If you or your funder prefer that the Contribution is made freely available online to non-subscribers immediately upon publication (gold open access), you may opt for the Contribution to be included in SAGE Choice, subject to payment of a publication fee. For further information, please visit SAGE Choice at http://www.sagepub.com/sagechoice.sp.

Additional Contributor Information

Contributors—Please complete the following two sections and return with your signed Agreement. Please attach additional pages if needed.

I. Financial Support Disclosure and Declaration of Potential Conflicts of Interest:

Contributors certify that the following given answers are a thorough and accurate representation of (a) the financial support received by each Contributor, either directly or indirectly, that relates to the Contribution or its underlying research and (b) all potential conflicts of interest of each Contributor. The following questions should be fully answered with respect to each Contributor:

1. Have any Contributors, either directly or indirectly, received financial support of any kind from a government, charitable, academic, or other public funding agency related to Contributor's research or authorship of the Contribution? **Yes** X **No** ______

If "Yes," please identify the Grant number(s) under which such funding was received: ESRC Award Reference 1012224.

And please describe all such forms and sources of the government and/or other public funding agency support:
PhD Studentship Grant from the Economic and Social Research Council intthe UK

2. Have any Contributors, either directly or indirectly, received financial support, from any commercial or corporate entities (including, but not limited to pharmaceutical companies), related to Contributors' research, authorship, or promotion of the Contribution? **Yes**______ **No** X

If "Yes," please describe all such forms and sources of commercial/corporate financial support related to the Contribution, including, for each Contributor: (a) the name of the supporting entity, (b) the interests it represents and/or its product discussed or alluded to within the Contribution, and (c) a description of the provided support:

3. Do any Contributors have any commercial or financial involvements that might present an appearance of a conflict of interest related to the Contribution (e.g., employment, consulting, board membership, current patents or patent applications, or investment in entities with an interest in the Contribution or its underlying research)? Please consider the past five years as well as current and prospective involvements. **Yes**______ **No** X

If "Yes," please identify and describe all such commercial or financial involvements:

4. Have any Contributors signed agreements with sponsors of the research reported in the Contribution that place any requirements, such as preventing the publication of both positive and negative results or forbidding Contributors from publishing the research without the prior approval of the sponsor, on their publication of the research findings? **Yes**______ **No** X

If "Yes," please identify all sponsor agreements for each Contributor, including a description of any publication requirements:

5. Have any Contributors, directly or indirectly, had any other relationships or involvement, whether or not of a financial nature, that may have influenced, or that may give the appearance of potentially influencing, the authorship or content of the Contribution? Please consider the past 5 years as well as current and prospective involvements. **Yes**______ **No** X

If "Yes," please describe all such support, relationships, or involvements:

SAGE reserves the right to include any information provided by Contributors in the preceding sections within the published Contribution as part of the Financial Disclosure Statement. If no conflicts of interest are declared, then the following statement may be printed with the Contribution: "*The Authors declared that they had no conflicts of interests in their authorship and publication of this Contribution.*"

II. Additional Items:

1. The Contribution contains third-party material and/or personal images requiring permission. (Please forward all permission agreements to the Editorial Office within five [5] days after signing this Agreement). **Yes**______ **No** X

TERMS OF AGREEMENT

1. Warranties; Indemnification

Contributors, jointly and severally, warrant and represent that (a) all Contributors have the full power and authority to enter into and execute this Agreement and to license the rights granted herein and that such rights are not now subject to prior assignment, transfer, or other encumbrance; (b) the Contribution is the original work of Contributors (except for copyrighted material owned by others for which written permission has been obtained), has not been previously published in any form (except for any previous public distribution of the Contribution, which has been disclosed in writing to the Editor), and has been submitted only to the Journal; (c) the Contribution and Supplemental Materials, if any, do not infringe the copyright or violate any proprietary rights, rights of privacy or publicity, or any other rights of any third party, and do not contain any material that is libelous or otherwise contrary to law; (d) all statements and presentation of data in the Contribution and Supplemental Materials, if any, asserted as factual are either true or based on generally accepted professional research practices and no formula or procedure contained therein would cause injury if used in accordance with the instructions and/or warnings included in the Contribution; and (e) any studies on which the Contribution is directly based were satisfactorily conducted in compliance with the governing Institutional Review Board (IRB) standards or were exempt from IRB requirements. In the event that any of the foregoing warranties or representations are breached, Contributors, jointly and severally, shall indemnify and hold harmless the Editor and SAGE, their affiliates, assigns, and licensees, against any losses, liabilities, damages, costs and expenses (including legal costs and expenses) arising from or resulting out of any claim or demand of any kind relating to such breach.

2. Supplemental Materials

Supplemental Materials, as used in this Agreement, means all materials related to the Contribution, but not considered part of the typeset Contribution as published in the Journal, provided to SAGE by Contributors. Supplemental Material may include, but is not limited to, data sets, audio-visual interviews and footage including podcasts (audio only) and vodcasts (audio and visual), appendices, and additional text, charts, figures, illustrations, photographs, computer graphics, and film footage. Contributors' grant of a non-exclusive right and license to SAGE for these materials in no way restricts re-publication of the Supplemental Materials by Contributors or anyone authorized by Contributors.

3. U.S. Government Works

If the Contribution is a U.S. Government work, then Contributors hereby certify that all Contributors were officers or employees of the United States Government at the time the Contribution was prepared and that the Contribution was prepared by Contributors as part of their official government duties. SAGE acknowledges that under the U.S. Copyright Act of 1976, as amended, United States copyright protection is not available for U.S. Government works, which are considered to be in the public domain in the United States. SAGE acknowledges that Contributors' execution of this Agreement documents their permission to SAGE to publish the Contribution and signifies that Contributors agree with all other terms of this Agreement, but does not convey an exclusive license to SAGE to publish the Contribution. SAGE acknowledges that contributors of U.S. Government works may not be able to accept complimentary copies of their contribution, or may only accept the complimentary copies on behalf of their employing agency, and SAGE will follow Contributors' preference with respect to providing complimentary copies.

4. Works Prepared Under U.S. Government Contract or Grant

If the Contribution was prepared under a U.S. Government contract or grant, SAGE acknowledges that the United States Government reserves a royalty-free, non-exclusive, and irrevocable right to reproduce, publish, or otherwise use the Contribution for official United States Government purposes only, and to authorize others to do so, if the U.S. Government contract or grant so requires. However, such works will not be considered U.S. Government works, as described previously. Upon request, Contributors will provide SAGE with the contract number of the U.S. Government contract or grant and/or copy of the contract.

5. Contributors' Credit

Each Contributor will receive credit as an author of the Contribution when it is published in the Journal; the form and placement of the credit will be determined by SAGE. Corresponding Contributor is responsible for providing the name and contact information of each Contributor to the Editor and/or SAGE in order to ensure accurate credit. Each Contributor grants SAGE and its licensees and grantees the right to use such Contributor's name, likeness, biographical information, and professional credits on copies of the Contribution and the Journal and in connection with the exercise of any other of SAGE's rights granted hereunder and in advertising, marketing, and promotion in connection therewith, and to grant such rights to any licensees or assigns of SAGE's rights hereunder.

6. Copyediting; Proofreading; Color Images

The Editor and/or SAGE may copyedit the Contribution and Supplemental Materials, if any, for clarity, brevity, accuracy, grammar, word usage, and style conformity and presentation as the Editor and/or SAGE deems advisable for production and publication in the Journal. Corresponding Contributor shall proofread proofs of the Contribution and indicate any proposed corrections or other changes and their timely return to SAGE as directed, with time being of the essence. SAGE may charge to Contributors the cost of making extensive text changes in proofs requested by Corresponding Contributor at a rate of $2.00 per line. If corrected proofs are not timely returned, SAGE may proceed with the publication of the Contribution as it deems appropriate. In the event Contributor desires to include color images in the Contribution as published in the print edition of the Journal, Contributor shall notify Editor, and Editor will advise Contributor of any additional charges, at the Journal's then-current rate, that may apply. Color images may be included in Contribution as published in the online edition of the Journal at no charge.

7. Termination

This Agreement must be signed by or on behalf of all the copyright holders in the Contribution as a condition of publication. SAGE makes no guarantee that the Contribution will be published in the Journal. If for any reason the Contribution is not published in the Journal, then all rights in the Contribution granted to SAGE shall revert to Contributors and this Agreement shall be of no further force and effect, and neither SAGE nor Contributors will have any obligation to the other with respect to the Contribution.

8. Dispute Resolution

In the event a dispute arises out of or relating to this Agreement, the parties agree to first make a good-faith effort to resolve such dispute themselves. Upon failing, the parties shall engage in non-binding mediation with a mediator to be mutually agreed on by the parties. Any controversy or claim arising out of or relating to this Agreement, or the breach thereof, which the parties cannot settle themselves or through mediation, shall be settled by arbitration in Ventura County, California, and administered by the American Arbitration Association in accordance with its then-existing Commercial Arbitration Rules. The award rendered by the arbitrator or arbitrators shall be final, and judgment may be entered upon it in accordance with applicable law in any court having jurisdiction thereof. The parties consent to, and waive any right to object to, jurisdiction with respect to the resolution of disputes hereunder in Ventura County, California. In any legal action or other proceedings (including arbitration proceedings) between the parties, the prevailing party shall be entitled to recover from the non-prevailing party all reasonable costs and expenses incurred in such action or proceeding, including without limitation, reasonable attorneys' fees and costs.

9. Governing Law

The validity, interpretation, performance and enforcement of this Agreement shall be governed by the laws of the State of California without regard to its conflicts of laws provisions which would give rise to the application of domestic substantive law of any other jurisdiction. Subject to Section 7, each party hereby consents to the jurisdiction and venue of the courts of the State of California located in Ventura County and of the U.S. District Court for the Central District of California.

10. Counterparts; Facsimile

This Agreement may be executed in counterparts, each of which shall be deemed the original, all of which together shall constitute one and the same instrument. A faxed copy or other electronic copy of this Agreement shall be deemed an original.

11. Electronic Signature Authorization

This transaction may be conducted by electronic means and the parties authorize that their electronic signatures act as their legal signatures of this Agreement. This Agreement will be considered signed by a party when his/her/its electronic signature is transmitted. Such signature shall be treated in all respects as having the same effect as an original handwritten signature. (Contributors are not required to conduct this transaction by electronic means or use an electronic signature, but if Contributors choose to do so, then Contributors' authorization is hereby given pursuant to this paragraph.)

12. Modification; Entire Agreement; Severability

No amendment or modification of any provision of this Agreement shall be valid or binding unless made in writing and signed by all parties. This Agreement constitutes the entire agreement between the parties with respect to its subject matter, and supersedes all prior and contemporaneous agreements, understandings, and representations. The invalidity or unenforceability of any particular provision of this Agreement shall not affect the other provisions, and this Agreement shall be construed in all respects as if any invalid or unenforceable provision were omitted.

CONTRIBUTORS' RESPONSIBILITIES

SAGE is sympathetic to the needs of scholars to include other copyrighted material in their Contribution and is happy to provide guidance on this. Responsibility for obtaining permission for any copyrighted material that cannot be included under the Fair Use doctrine rests with Contributors.

If the Contribution includes material for which Contributors do not hold the copyright, Contributors are responsible for submitting with the Contribution the written permission from the copyright holder of the material to include and reproduce the material within the Contribution. The permission must cover all media and all languages throughout the world in perpetuity. Contributors are responsible for the payment of such permissions.

Please note: Whether the material is being used with permission, or on the basis that it falls under "fair dealing" or "fair use," full citation for the copyright holder and original publication of the material must be included with the submission.

CONTRIBUTORS' RIGHTS

1. You retain the copyright in your Contribution.
2. You may do whatever you wish with the version of the Contribution you submitted to the Journal (version 1).
3. Once the Contribution has been accepted for publication, you may post the accepted version of the Contribution (version 2) on your own personal website, your department's website, or the repository of your institution without any restrictions.
4. You may not post the accepted version of the Contribution (version 2) in any repository other than those listed above (i.e., you may not deposit in the repository of another institution or a subject repository) until 12 months after publication of the Contribution in the Journal.
5. You may use the published Contribution (version 3) for your own teaching needs or to supply on an individual basis to research colleagues, provided that such supply is not for commercial purposes.
6. You may use the Contribution (version 3) in a book you write or edit any time after publication in the Journal.
7. You may not post the published article (version 3) on a website or in a repository without permission from SAGE.
8. When posting or re-using the Contribution, please provide a link to the appropriate DOI for the published version of the Contribution on SAGE Journals (http://online.sagepub.com)

All commercial and any author re-use of the Contribution not specified above should be referred to SAGE. More information can be found at http://www.sagepub.com/journalsPermissions.nav.

SAGE will directly, or through a third-party intermediary, provide the Corresponding Contributor of the Contribution with an electronic copy of the Contribution.

SAGE'S USE OF THE CONTRIBUTION

Although Contributors have retained the copyright in the Contribution, Contributors have granted SAGE an exclusive license to exercise the rights under copyright. This helps ensure protection against infringement of copyrighted material through breach of copyright or piracy anywhere in the world. It also ensures that requests from third parties to reprint or reproduce all or any part of the Contribution are handled efficiently many years after publication of the Contribution, ensuring the ability of the Contribution to receive broad dissemination.
